# Psychiatric Manifestations of Hypoglycemia in an Adolescent: A Case Report

**DOI:** 10.1002/ccr3.70773

**Published:** 2025-08-08

**Authors:** Mulugeta Sitot Shibeshi, Abayneh Girma Tolcha, Tigist Zerihun

**Affiliations:** ^1^ Pediatric Neurology Unit, Department of Pediatrics and Child Health Hawassa University College of Medicine and Health Sciences Hawassa Ethiopia; ^2^ Department of Psychiatry Hawassa University College of Medicine and Health Sciences Hawassa Ethiopia; ^3^ Child and Adolescent Psychiatry Division, Department of Psychiatry, St Paul Millennium Medical College Addis Ababa Ethiopia; ^4^ Neurodiversity Center Ethiopia Addis Ababa Ethiopia

**Keywords:** case report, fluoxetine, hypoglycemia, misdiagnosis, psychiatric manifestations

## Abstract

Psychiatric manifestations of hypoglycemia are rarely reported and can lead to misdiagnosis and mistreatment. When patients with diabetes present with psychiatric symptoms, hypoglycemia should be considered a possible cause before prescribing psychotropic drugs. Fluoxetine can induce hypoglycemia and its associated psychiatric symptoms.

## 1 Introduction

Hypoglycemia is a blood glucose level of less than 70 mg/dL (3.9 mmol/L) and is a frequently observed complication of diabetes treatment [1]. In the absence of impaired hypoglycemia awareness, a blood glucose level below 70 mg/dL (3.9 mmol/L) triggers the release of counter‐regulatory hormones that result in adrenergic symptoms such as faintness, tremulousness, palpitations, hunger, diaphoresis, and nervousness. When hypoglycemia persists and the blood glucose level drops below 54 mg/dL (3.0 mmol/L), patients begin to manifest neuroglycopenic symptoms that include headache, confusion, impaired problem‐solving and concentration, ataxia, visual disturbances, motor weakness, hallucinations, and bizarre behavior [1]. If hypoglycemia continues without intervention, it can result in coma, permanent brain injury, or even death [2].

Psychiatric manifestations of hypoglycemia are rarely reported but can include symptoms such as acute psychosis [3], depression, insomnia, anxiety, irritability, difficulty concentrating, crying spells, phobias, forgetfulness, confusion, antisocial behavior, and suicidal tendencies [4].

Patients exhibiting these psychiatric symptoms may be misdiagnosed with a psychiatric illness that requires treatment [5]. Additionally, some medications prescribed for these presumed psychiatric conditions can worsen hypoglycemia. For example, fluoxetine ─ a medication often prescribed for depression, adjustment disorder, and other psychiatric illnesses─ has been shown to induce hypoglycemia [6, 7]. A review of preclinical and clinical studies found that fluoxetine increases insulin sensitivity, reduces hyperglycemia, and normalizes glucose homeostasis [8].

It is crucial for caregivers of patients with diabetes to recognize the psychiatric symptoms of hypoglycemia early. Delayed diagnosis and treatment with psychotropic medications that may exacerbate hypoglycemia can lead to severe complications, including irreversible neurologic damage or even death [2].

This case report underscores the need for the creation of awareness among clinicians, patients, and their caregivers about the psychiatric manifestations of hypoglycemia in individuals with diabetes. It also highlights the increased frequency of hypoglycemic episodes (and the associated psychiatric symptoms) that can occur with the use of fluoxetine. Additionally, it emphasizes the need for clinicians to exercise caution and minimize unnecessary exposure to psychotropic medications like fluoxetine for those living with diabetes.

## 2 Case History

A 17‐year‐old Ethiopian female adolescent was diagnosed with type 1 diabetes five months before her visit to the psychiatry department of Hawassa University Comprehensive Specialized Hospital. At that time, she was using a combination of regular insulin and neutral protamine Hagedorn (NPH) insulin injections. Her total daily insulin dose was 1 IU/kg/day, with two‐thirds being NPH and one‐third regular insulin. She administered 2/3 of her total daily insulin dose in the morning and 1/3 in the evening. She was injecting herself regularly with occasional parental supervision. She had three main meals and two snacks in a day, and she did not skip any meals. Although she was unable to measure her blood glucose regularly due to a shortage of test strips, her fasting blood glucose levels ranged from 54 mg/dL to 180 mg/dL, while her evening levels ranged from 70 mg/dL to 250 mg/dL. Her most recent glycated hemoglobin (HbA1c) level was 9%.

Ten days before her presentation to the psychiatry department, she experienced episodes of early morning altered behavior that included agitation, shouting, and bizarre motor activity. Although these abnormal behaviors lasted several minutes, they were not accompanied by loss of consciousness or convulsions. After having breakfast, her symptoms gradually improved, and she did not experience these behaviors during the rest of the day.

She is the first child in a family of five, with 2 siblings. She is a grade 11 student with good academic performance, and she is well‐loved by both her friends and teachers. The patient has no previous personal or family history of psychiatric illness. Both of her parents are government employees and have experienced marital discord that began a couple of weeks before the onset of her current symptoms.

During the first few episodes of her symptoms, her caretakers believed she was possessed by an evil spirit. They sprinkled holy water on her and prayed for her mental health to improve. However, her episodic early morning bizarre behavior persisted for several days, prompting her parents to seek help from a psychiatrist. The psychiatric assessment revealed symptoms such as lack of interest, depressed mood, poor sleep, excessive time spent at home, and diminished appetite following her diabetes diagnosis. Both systemic and neurologic examinations were unremarkable. Renal, liver, and thyroid function tests were within normal limits.

## 3 Differential Diagnosis

Given the stress of managing her diabetes and the recent marital issues involving her parents, she was given a preliminary diagnosis of conversion disorder for her new onset bizarre behavior. Additionally, because of the presence of symptoms suggestive of depression, she was also diagnosed with major depressive disorder (MDD). She was prescribed fluoxetine at a dose of 20 mg to be taken once daily in the morning, along with plans for psychotherapy. Unfortunately, her episodic early morning abnormal body movements and shouting increased in frequency shortly after starting fluoxetine, escalating from one or two episodes per week to almost daily occurrences.

One morning, while she was experiencing psychiatric symptoms, her caretakers measured her blood glucose and found it to be low (36 mg/dL). She was given sugar and honey, and her symptoms improved quickly when her blood glucose level normalized. A few days later, she had another episode of bizarre behavior associated with a low blood glucose level (24 mg/dL), which also improved after correcting the hypoglycemia. Subsequently, she was taken to the diabetes clinic, where her psychiatric symptoms were attributed to hypoglycemia. As a result, she was provided with diabetes self‐management education and support, her insulin dosage was reduced by 15%, and her caretakers were advised to provide her with sugar or other sugar‐containing foods and drinks whenever she experiences similar symptoms. At the psychiatry clinic, the diagnosis of conversion disorder was dropped, the fluoxetine was discontinued, and psychotherapy was planned for her depression.

The patient might have experienced diabetes distress from the ongoing demands of self‐care and a fear of complications [9]. However, she was not formally evaluated for the presence of diabetes distress. This represents a significant gap in the care of children with diabetes in this setting that needs attention.

## 4 Conclusion and Results (Outcome and Follow‐Up)

Hypoglycemia was the cause of the bizarre behaviors. Fluoxetine was discontinued since it worsened the hypoglycemia and its associated psychiatric symptoms. The patient was referred for psychotherapeutic intervention to address her depression. During follow‐up visits for more than a year, the patient avoided hypoglycemia and did not experience any further psychiatric symptoms.

## 5 Discussion

This case highlights the psychiatric manifestations of hypoglycemia in an adolescent with type 1 diabetes, which were initially misdiagnosed as a conversion disorder. The fluoxetine prescribed for her comorbid major depressive disorder (MDD) resulted in more frequent episodes of hypoglycemia and its associated psychiatric symptoms. The psychiatric symptoms improved with the correction of the hypoglycemia.

Children and adolescents with diabetes are at risk of developing psychiatric problems such as depression, mood disorders, disruptive behavior disorders, anxiety, and eating disorders [10, 11]. Hence, they should be regularly screened for mental health problems as psychiatric disorders are highly prevalent in this population group [12]. In our patient, neither the parents nor the health care professionals providing diabetes care were aware of the presence of MDD until she had a psychiatric evaluation for the bizarre behavior associated with hypoglycemia.

The likely cause of hypoglycemia was the high dose of insulin (1 IU/kg/day) that the patient was taking during the honeymoon phase of the disease when the body requires a relatively lower amount of insulin [13].

The brain primarily depends on glucose as its energy source, so hypoglycemia can have a detrimental effect on cognitive function [2]. The association between hypoglycemia and transient cognitive, affective, and somatic symptoms is well established [14]. Patients experiencing hypoglycemia may exhibit symptoms that resemble various psychiatric disorders, including acute psychosis [3], generalized anxiety disorder [15], catatonia [16], depression, and insomnia [4]. As a result, patients with hypoglycemia manifesting psychiatric symptoms might be misdiagnosed with a psychiatric disorder, and it is not uncommon for psychiatrists to expose them to psychotropic drugs when, in fact, the psychiatric symptoms can resolve with the correction of the hypoglycemia [5, 17].

Fluoxetine, a selective serotonin reuptake inhibitor, is commonly used to treat different psychiatric disorders, including depression, anxiety disorders, obsessive–compulsive disorder, and other mental disorders. Several reports have revealed that fluoxetine can induce hypoglycemia [6, 7]; increasing insulin sensitivity [18, 19] and reducing gluconeogenesis [19] are among the proposed mechanisms.

In the case described, the caretakers were unaware of the psychiatric symptoms associated with hypoglycemia. As a result, the patient's bizarre behavior was mistakenly attributed to possession by an evil spirit, a common belief in Ethiopia. During the initial episodes of the bizarre behavior, the blood sugar level was not measured due to a shortage of test strips. When test strips became available, the caretakers continued to use holy water and prayers, overwhelmed by her frightening symptoms. Initially, they were emotionally disturbed and did not have the calmness necessary to measure her blood glucose, which they would do routinely whenever test strips were available. After the initial episodes, they became accustomed to her symptoms and measured her blood glucose while she was symptomatic and found it low.

At the psychiatry clinic, hypoglycemia was not considered as the cause of the psychiatric symptoms because of the presence of multiple stressors that could explain her symptoms, which were initially attributed to conversion disorder. Additionally, the fluoxetine prescribed for her depression resulted in more frequent symptoms. The increased frequency of psychiatric symptoms is attributed to hypoglycemia caused by fluoxetine, as there was no change in the insulin dosage or the patient's feeding habits. However, induction of hypoglycemia may not be the only mechanism for the development of the bizarre behavior, as there have been reports of psychosis associated with the use of fluoxetine that are unrelated to hypoglycemia [20, 21]. The presumed mechanisms for the psychiatric symptoms include a 5—hydroxytryptamine 3 (5‐HT3)—mediated dopamine release, beta‐noradrenergic receptor downregulation, or gamma‐aminobutyric acid (GABA) B‐receptor upregulation acting in the vicinity of the ventral basal ganglia [21]. However, the psychiatric symptoms that emanate from these mechanisms tend to resolve slowly after discontinuing fluoxetine, which is related to its long half‐life, lasting up to 16 days for its active metabolite [22].

In our case, the timing and duration of the psychiatric manifestations, along with their immediate improvement after ingestion of food and correction of the hypoglycemia, indicate a temporal relationship between the occurrence of hypoglycemia and the psychiatric symptoms.

In conclusion, when patients with diabetes present with psychiatric symptoms, hypoglycemia should be considered a possible cause before prescribing psychotropic drugs. In addition, clinicians, patients, and caregivers should be aware of hypoglycemia associated with fluoxetine use and the need for careful monitoring of blood sugar in patients with diabetes who are taking fluoxetine.

## Author Contributions

**Mulugeta Sitot Shibeshi:** conceptualization, data curation, investigation, resources, supervision, writing – original draft, writing – review and editing. **Abayneh Girma Tolcha:** conceptualization, data curation, investigation, resources, writing – review and editing. **Tigist Zerihun:** conceptualization, data curation, investigation, writing – review and editing.

## Ethics Statement

Written informed consent was obtained from the patient's mother for the publication of the anonymized information of her daughter. A verbal assent was also obtained from the patient when her mental health condition improved. Our institution does not require ethical approval for reporting individual cases or case series.

## Conflicts of Interest

The authors declare no conflicts of interest.

## Data Availability

The data that support the findings of this study are available from the corresponding author upon reasonable request.
